# CpG incorporated DNA microparticles for elevated immune stimulation for antigen presenting cells†

**DOI:** 10.1039/c7ra13293j

**Published:** 2018-02-09

**Authors:** Heejung Jung, Dajeong Kim, Yoon Young Kang, Hyejin Kim, Jong Bum Lee, Hyejung Mok

**Affiliations:** Department of Bioscience and Biotechnology, Konkuk University 120 Neungdong-ro, Gwangjin-gu Seoul 05029 Republic of Korea hjmok@konkuk.ac.kr; Department of Chemical Engineering, University of Seoul 163 Seoulsiripdaero, Dongdaemun-gu Seoul 02504 Republic of Korea jblee@uos.ac.kr

## Abstract

As emerging evidence supports the immune stimulating capability of the CpG oligodeoxynucleotides (ODN), CpG-based adjuvants have been widely used. For efficient induction of immune responses, current issues affecting the use of nucleic acid-based adjuvants, *e.g.* stability in physiological conditions, delivery to immune cells, and successful release within the phagolysosome, should be addressed. Here, we present CpG-based DNA microparticles (DNA-MPs) fabricated by complementary rolling circle amplification (cRCA) as adjuvants for enhancing immune response and production of selective antibody production. Using cRCA method, the sizes of CpG-based DNA-MPs were finely controlled (0.5 and 1 μm) with superior and provided mismatched single stranded form of CpG ODN region for specific cleavage site by DNase II within the phagolysosome. Fabricated CpG-based 1 μm DNA-MPs (DNA-MP-1.0) were successfully internalized into primary macrophages and macrophage cell line (RAW264.7 cells), and elicited superior cytokine production *e.g.* TNF-α and IL-6, compared to conventional CpG ODNs. After *in vivo* administration of DNA-MP-1.0 with model antigen ovalbumin (OVA), significantly elevated OVA-specific antibody production was observed. With this in mind, DNA-MP-1.0 could serve as a novel type of adjuvant for the activation of macrophages and the following production of selective antibodies without any noticeable toxicity *in vitro* and *in vivo*.

## Abstract 2

As emerging evidence supports the immune stimulating capability of the CpG oligodeoxynucleotides (ODN), CpG-based adjuvants have been widely used.

## Graphical abstract

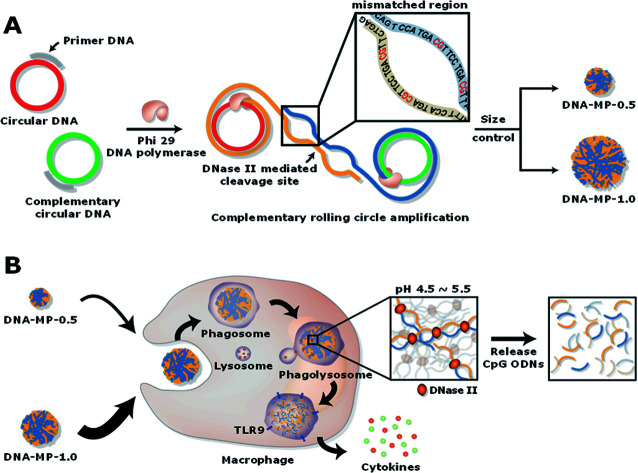

## Introduction

Efficient and selective immune stimulation against disease-related antigens have been noticed as emerging therapeutic approaches for the treatment of cancers, allergies, and immune-related diseases.^1,2^ Accordingly, diverse immune-eliciting biomaterials including alum salts, monophosphoryl lipid a (MPLA), and liposomes have been investigated to provide efficient induction of immune responses with a low dose of vaccine antigens, fast immune responses, induction of a variety of antibodies, and wide range of humoral and cell-mediated immune responses.^3,4^ Still, several antigens showed poor stimulation and antibody production by current adjuvant systems.^5,6^ Thus, safe and selectively strong immune stimulators need to be developed. As a novel type of immune stimulating adjuvant system, CpG oligodeoxynucleotide (ODN), a ligand for Toll-like receptor-9 (TLR-9), has been noted *in vivo*.^7,8^ Unlike methylated CpG sequences in mammalian cells, unmethylated CpG sequences in bacteria could be recognized by TLR-9, which triggers strong humoral and cell-mediated immune responses *via* macrophages, dendritic cells, NK cells, and B lymphocytes.^8–10^ However, CpG ODN with its negative charge hinders efficient internalization into cells with poor serum stability, which limits efficient induction of immune responses for antigen presenting cells (APCs).^11^ To improve the stability of CpGs, chemical modifications and double-stranded CpG structures could be considered. However, chemically modifying CpG ODN, with for example phosphorothioate modification, showed severe side effects in the body, such as renal damage, despite improved enzyme stability and efficient cytokine induction, compared to natural ODN with phosphodiester bonds.^10,12^ In addition, single stranded CpG-containing DNA has a much higher binding affinity to TLR-9 and allows facile dimerization of TLR-9 for activation, than that of double stranded CpG-containing DNA.^13^ Accordingly, particle-based delivery systems have been studied to protect CpG ODN from serum proteins and enzymes and to improve intracellular delivery without any loss of biological activity and side effects.^14^

A wide range of nucleic acid-based structures *e.g.* particles, conjugates, and origami have been considered as carriers for drug delivery and imaging, as well as immune-therapeutics themselves.^15–18^ Interestingly, bulky and complex DNA structures have elicited strong immune responses for macrophages.^19–21^ For example, dendrimer-like DNA nanostructures with a size of below 50 nm have exhibited much better cytokine induction for RAW264.7 cells than Y-shaped CpG DNA structures and conventional CpG ODN.^19^ Similarly, CpG based nanoparticles with a size of ∼300 nm, synthesized by rolling circle amplification (RCA), have shown significant cytokine induction and biocompatibility without additive carriers *in vitro*.^20^ However, there has been no study on effective immunostimulation by controlling the size of CpG-based particles or designing the sequence of the template for the efficient release of CpG ODNs.

In this study, we have produced complementary RCA (cRCA)-based DNA microparticles composed of repeated CpG (DNA-MPs) with well-controlled size distribution and a mismatched double stranded region between CpG sites for specific cleavage by DNase II. 1 μm DNA-MP (DNA-MP-1.0) and 0.5 μm DNA-MP (DNA-MP-0.5) were fabricated and examined by SEM analysis, and studies on serum stability and release studies were undertaken in the presence of DNase II. Intracellular uptake of DNA-MPs was investigated using two types of macrophages, RAW264.7 cells and bone marrow-derived macrophages (BMDMs) and was analyzed using *in vivo* imaging instruments (IVIS), fluorescence-activated cell sorting (FACS) analysis, and confocal microscopy. A cytokine release study, *e.g.* using TNF-α and IL-6, was performed on RAW264.7 cells and BMDMs and was analyzed by enzyme-linked immunosorbent assay (ELISA). After intramuscular injection of DNA-MPs with model antigen ovalbumin (OVA) *in vivo*, the extent of antibody production was quantitatively analyzed by ELISA.

## Materials and methods

### Materials

CpG 1826 was selected from diverse model CpG ODN sequences^22^ and was obtained from Bioneer (Daejeon, South Korea) as shown in Table 1. Another five types of ODNs were purchased from Integrated DNA Technologies (Coralville, Iowa, USA). T4 DNA ligase was from Promega (Madison, WI, USA). The dNTP mix, Phi 29 polymerase, and Cy5-labeled dCTP (Cy5-dCTP) were purchased from Epicentre (Madison, WI, USA), Lucigen (Middleton, WI, USA), and GE Healthcare (Chicago, IL, USA), respectively. Deoxyribonuclease II (DNase II) from porcine spleen, red blood cell (RBC) lysis buffer, ovalbumin (OVA), and Complete Freund's Adjuvant (CFA) were obtained from Sigma-Aldrich (St. Louis, MO, USA). Dulbecco's modified Eagle's medium (DMEM), Roswell Park Memorial Institute (RPMI) 1640 medium, penicillin/streptomycin (P/S), and fetal bovine serum (FBS) were purchased from Gibco BRL (Grand Island, NY, USA). Mouse granulocyte-macrophage colony-stimulating factor (mGM-CSF) were purchased from Peprotech (Seoul, Korea). Fluorescence labeled anti-EEA1 antibodies were obtained from Abcam (Cambridge, USA). Mouse IL-6 and TNF-α ELISA kits were purchased from BD Bioscience (Franklin Lake, NJ, USA). Mouse anti-ova IgG was obtained from Alpha Diagnostic International (San Antonio, TX, USA). C57BL/6 mice (female, 6 weeks old) were purchased from Orient Bio Inc. (Seongnam, Korea). POPO-3 iodide was obtained from Invitrogen (Carlsbad, CA, USA).

**Table 1:** Sequence information of naked CpG and linear DNAs for generating DNA-MPs. Naked CpG DNA, primers, linear DNAs for CpG, GpC, and their complementary strands. Blue: hybridization sites with primers; red: 20-base long CpG ODN; underlined red: CpG or GpC dinucleotide sites

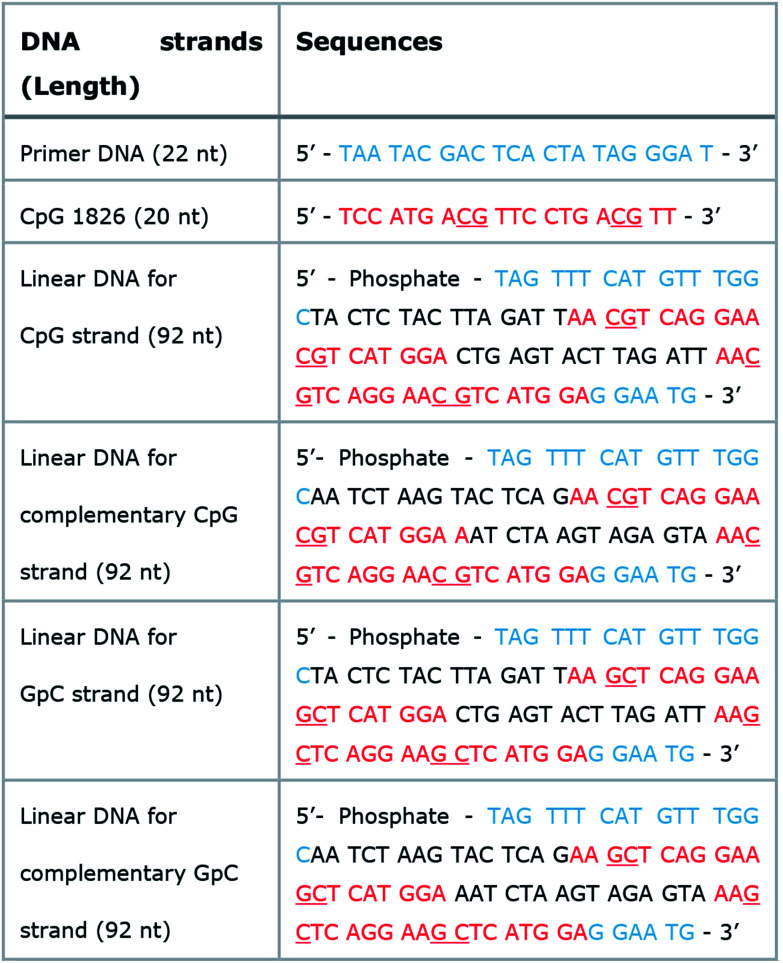

### Enzymatic synthesis of CpG based DNA-MPs using cRCA

Phosphorylated 92 base-long linear DNA and 22 base-long primer DNA (10 μM) were first mixed in nuclease-free water and annealed by heating at 95 °C for 2 min, followed by gradual cooling to 25 °C for more than 1 h using a thermal cycler (Bio-Rad). To ligate the nick in the circularized DNA, the mixed solution was incubated at room temperature overnight with T4 DNA ligase (0.3 U μl^−1^) in reaction solution (30 mM of Tris–HCl, 10 mM of MgCl_2_, 10 mM of DTT (dithiothreitol) and 1 mM of ATP (adenosine triphosphate)). For cRCA of DNA-MP-1.0, two circular DNAs (0.5 μM) were incubated with a dNTP mix (2 mM), Phi 29 polymerase (1 U μl^−1^) and reaction buffer (100 mM of Tris–HCl, 20 mM of (NH_2_)_2_SO_4_, 8 mM of DTT and 20 mM of MgCl_2_) at 30 °C for 20 h. In addition, 1.0 μM of each circular DNA with 0.8 mM or 2 mM of dNTP were used to generate the DNA-MP-0.5 and 0.8 μm DNA-MP (DNA-MP-0.8), respectively. The final reactant was sonicated briefly to break up any aggregated particles. The sonicated solution was centrifuged at 6000 rpm for 5 min and washed with nuclease free water several times to collect the particles from the reaction mixture. For the fluorescence experiments, Cy5-dCTP (0.08 mM) was added to the reaction mixture. Characterization of CpG based DNA-MPs: three types of DNA-MPs, DNA-MP-0.5, DNA-MP-0.8 and DNA-MP-1.0 (20 ng) were loaded onto a silicon wafer for scanning electron microscopy (SEM) and Lacey film for transmission electron microscopy (TEM). S-4300 (Hitachi) field emission scanning electron microscopy (FE-SEM) and JEM-2100F (JEOL) TEM were used to obtain high resolution images of the DNA-MPs and TEM-based energy dispersion X-ray (EDX) was used to analyze the chemical composition of DNA-MP-0.5 and DNA-MP-1.0.

### Release of CpGs from DNA-MPs

DNA-MP-1.0 (3 μg) was incubated with DNase II at different concentrations of enzymes (0, 2, 5, 10, and 50 U ml^−1^) in two types of buffer solution, sodium acetate buffer (pH 4.8) and PBS solution (pH 7.2) containing MgCl_2_ (0.8 mM) and NaCl (20 mM) at 37 °C for 4 h. After the enzyme reaction, samples were loaded onto 3.0% agarose gel and electrophoresis was carried out at 95 V for 90 min. The DNAs were stained with ethidium bromide for visualization. After DNase II enzyme reaction of DNA-MP-1.0 (3 μg) at an enzyme concentration of 50 U ml^−1^ for 4 h, particles were observed by SEM S-4300 (Hitachi).

### Serum stability of CpG based DNA-MPs

Two types of DNA-MP, DNA-MP-0.5 and DNA-MP-1.0, (3 μg) were incubated with 10% fetal bovine serum (FBS) for predetermined time intervals (0, 10 min, 1 h, and 24 h). Morphologies of the DNA-MPs were analyzed by SEM. Average diameters of the DNA-MPs were assessed based on SEM images using ImageJ software. More than 30 particles were analyzed to determine the average diameter of the particles.

### Cellular uptake of the DNA-MPs

To visualize the two types of DNA-MPs, DNA-MP-0.5 and DNA-MP-1.0 were stained with POPO-3 dye, according to the manufacturer's protocol. Briefly, the two types of DNA-MPs (30 μg) in deionized water (DW) were mixed with POPO-3 dye (100 μM) in 1× TAE buffer. The resulting solution was incubated for 1 h at 25 °C. Free POPO-3 dye was removed by centrifugation at 5000 rpm for 5 min at 4 °C and washed with DW four times. After purification, the amounts of DNA-MPs were analyzed by UV spectrophotometer at a wavelength of 260 nm, as previously reported.

RAW264.7 cells (murine macrophage cell line) were maintained in DMEM supplemented with 10% FBS, 100 U ml^−1^ penicillin, and 100 μg ml^−1^ streptomycin at 37 °C in a humidified atmosphere of 5% CO_2_. Cells were plated in 6-well plates at a density of 6 × 10^5^ cells per well 24 h prior to treatment. POPO-3 labeled DNA-MP-0.5 and DNA-MP-1.0 (2 μg) were added to the cells for 4 h in serum free media. For cytochalasin B treatment, cells were washed with PBS solution and cytochalasin B (20 mM) in serum free DMEM, were added to cells and incubated for 30 min. DNA-MPs (2 μg) were added to cells for 4 h in serum free media in the presence of cytochalasin B solution (10 mM). After incubation, cells were washed three times with PBS solution containing 5% FBS and isolated. Internalized POPO-3 labeled DNA-MP in isolated live cells were quantitatively analyzed by an IVIS instrument (Caliper Life Sciences Lumina II, Hopkinton, MA) at excitation and emission wavelengths of 535 and 583 nm, respectively.

Bone marrow-derived macrophages (BMDMs) were prepared as previously described.^23^ Briefly, bones of ICR mice (female, 6 weeks) were flushed with RPMI media containing 10% FBS to isolate murine macrophage progenitor cells from the bone marrow. The isolated cells were spun down by centrifugation at 1500 rpm for 5 min at 4 °C and incubated with RBC lysis buffer. After washing with media, cells were resuspended in a RPMI 1640 medium containing murine colony-stimulating factor (mGM-CSF; 10 ng ml^−1^) and cultured for 7 days. Adherent cells were gently scraped with a cell lifter, and 6 × 10^5^ cells were plated in 6-well plates. After 24 h, cells were stained with PE-labeled F4/80 antibodies and FITC-labeled CD-206 antibodies for FACS analysis. For particle treatment, BMDM cells were plated in 4-well chambers at a cell density of 1 × 10^5^ cells per well. Cells were treated with POPO-3 labeled DNA-MP-0.5 and DNA-MP-1.0 at a concentration of 2 μg ml^−1^ for 4 h. After removing the DNA-MPs, fresh RPMI medium with serum was added to cells and incubated for 1 h to remove nonspecific adsorption onto cells. After further washing with 1× trypsin–EDTA solution and PBS solution three times, cells were fixed with formaldehyde in PBS solution (3.7% v/v) for 1 h. Cells were mounted with DAPI solution and observed by confocal microscopy (LSM 710; Carl Zeiss, Oberkochen, Germany).

DNA-MP-1.0 was also covalently labeled with Cy5-dye (Cy5-DNA-MP-1.0), as follows. RAW264.7 cells were plated in 6-well plates at a density of 6 × 10^5^ cells per well 24 h prior to treatment. For cytochalasin B treatment, cells were washed with PBS solution and cytochalasin B (0 and 20 mM) in serum free DMEM, were added to cells and incubated for 30 min. Cy5-DNA-MP-1.0 (3.2 μg) was added to cells for 4 h in serum free media in the absence and presence of cytochalasin B solution (10 mM), respectively. After incubation, cells were washed three times with PBS solution containing 5% FBS and isolated. After fixation of cells with formaldehyde (3.7%) in PBS solution, cells were analyzed by FACS (Becton Dickson, Franklin Lakes, NJ, USA).

### Intracellular localization of DNA-MPs

RAW264.7 cells were plated in 4-well chambers at a density of 1 × 10^5^ cells per well 24 h prior to treatment. Cells were treated with Cy5-DNA-MP-1.0 at a DNA concentration of 3.2 μg ml^−1^ for 4 h. After incubation, the cells were washed three times and fixed with formaldehyde (3.7%) in PBS solution for 10 min at room temperature. For EEA1 staining, cells were incubated with Tween (0.1%) in PBS solution for 20 min. After permeabilization, the cells were treated with FITC-labeled anti-EEA1 antibodies in PBS solution with 10% FBS and 0.3 M glycine solution according to the manufacturer's protocol and incubated for 3 h. After staining, the cells were washed with PBS solution and mounted for confocal microscopy.

### Cell viability by the DNA-MPs

RAW264.7 cells were plated in 96-well plates at a density of 3 × 10^4^ cells per well 24 h prior to treatment. After overnight incubation, three different samples (free CpG, DNA-MP-0.5, and DNA-MP-1.0) in serum free media were treated to cells at different concentrations (0, 0.1, 1, 5, 10, 20 μg ml^−1^) for 4 h. After washing with DPBS solution, cell viability was assessed by CCK-8 assay, according to the manufacturer's protocol.

#### Cytokine release by the DNA-MPs

RAW264.7 cells were plated in 24-well plates at a density of 8 × 10^4^ cells per well 24 h prior to treatment. Cells were treated with DNA-MP-0.5 and DNA-MP-1.0 at different CpG concentrations (0.4, 0.8, 1.6, and 3.2 μg ml^−1^) for 8 h in serum media. After changing the incubation solution containing the DNA-MPs, cells were further incubated for 16 h in serum free media. The amount of TNF-α and IL-6 in the media were quantitatively analyzed by ELISA, according to the manufacturer's protocol.

For the selective cytokine release study using two types of DNA-MP-1.0, CpG-based and GpC-based DNA-MP-1.0, RAW264.7 cells and BMDMs were plated in 24-well plates at a density of 8 × 10^4^ cells per well 24 h prior to treatment, respectively. Cells were treated with CpG and GpC particles at a DNA concentration of 3.2 μg ml^−1^ for 8 h. After changing the media to fresh media and further incubation for 16 h, the amount of cytokines in the supernatant was analyzed by ELISA.

### Animal study

All animal care and experimental procedures were approved by the Animal Care Committee of Konkuk University. All animal procedures were performed in accordance with the Guidelines for Care and Use of Laboratory Animals of Konkuk University and approved by the Animal Ethics Committee of Konkuk University. Mouse immunization was performed as previously described with slight modification.^23^ Briefly, OVA (10 μg) samples with free CpG (25 μg), DNA-MP-1.0 (25 μg), and CFA (5 μl) were intramuscularly injected into C57BL/6 mice (female, 6–8 weeks old). PBS solution without OVA was also injected into mice as a control. After three times of repeated immunization on day 0, 14, and 24, sera were collected from mice on day 31 and analyzed by ELISA to quantitatively determine the level of OVA IgG using a mouse anti-OVA IgG ELISA kit.

## Results and discussion

### Synthesis of the CpG based DNA-MP

**Fig. 1:**
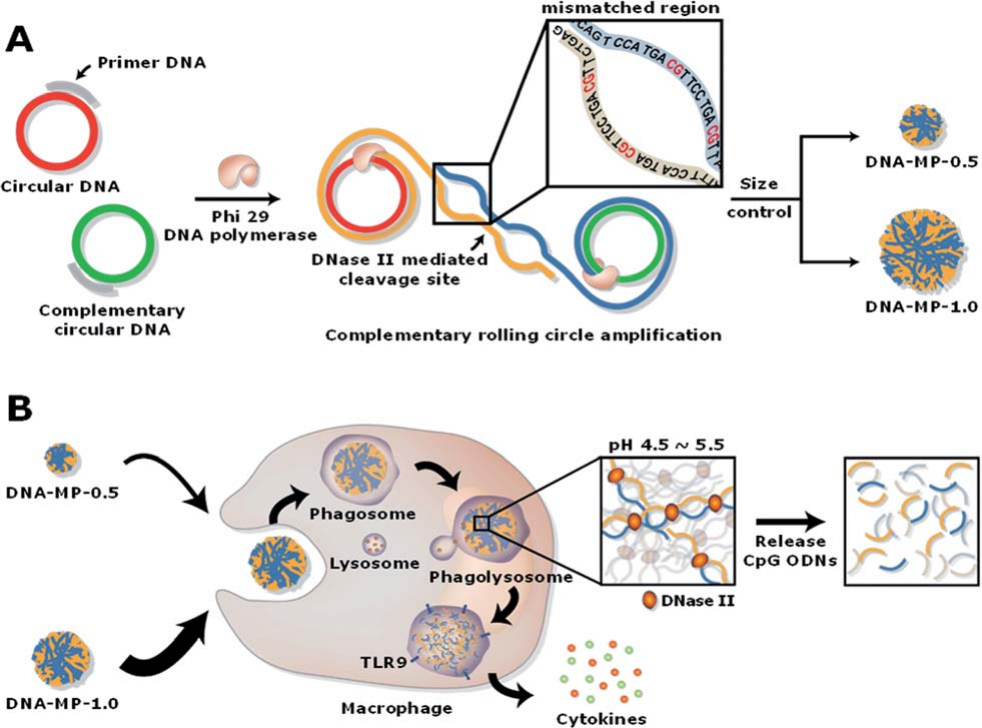
Schematic illustrations of the synthesis of the DNA-MPs and process for boosting immune response. (A) Complementary circular DNAs conjugated with primer DNAs replicating repeated CpG ODNs *via* complementary rolling circle amplification (cRCA) for producing self-assembled DNA-MPs with controlled sizes. (B) Phagocytosis of DNA-MPs by macrophages and subsequential release of CpG ODNs mediated by DNase II under acidic conditions, resulting in enhanced secretion of cytokines.

Micron sized particles have been mainly internalized into APCs, such as macrophages and dendritic cells *via* phagocytosis, which could be facilitated as targeting strategies for vaccines and adjuvants to APCs.^24^ In this study, homogeneous and micron sized particles with preferred phagocytic activities were fabricated using natural nucleic acids *via* cRCA. As shown in Fig. 1A, two types of circular DNA were designed to be partially complementary to each other, resulting in partially hybridized and long repeated double stranded DNA to encode CpG ODNs after polymerization and hybridization. The elongated DNA strands were entangled with each other and could be readily self-assembled to form immune-boosting DNA-MPs.^25,26^ To find the optimum size for cellular uptake and immune-boosting properties, the size of the DNA-MPs was finely controlled by adjusting the concentration ratio of the circular DNA to deoxyribonucleotide triphosphate (dNTP). The hybridized double stranded region of the DNA-MPs was expected to be cleaved by DNase II to release CpG ODNs efficiently from the mismatched region, due to the preference of DNase II to cleave double stranded regions than that of single strands under acidic conditions.^27^

Although the DNA-MPs are highly negative-charged, carrier-free CpG-based DNA-MPs were treated to macrophages for efficient immune stimulation without any additive polymers or lipid-based molecules to lessen the side effects. Fig. 1B shows that micro-sized particles engulfed by macrophages *via* phagocytosis could be readily dissociated by DNase II in the phagolysosome, releasing free CpG ODNs from the mismatched region for facile binding to TLR-9.^28^ Binding of CpG ODNs to functional TLR-9 in the phagolysosome allowed dimerization of TLR-9, which could recruit myeloid differentiation factor 88 (MyD88), IL-1R-associated kinase (IRAK), and tumor necrosis factor receptor-associated factor 6 (TRAF6). These intracellular pathways trigger cytokine induction and activation of macrophages *via* mitogen-activated kinases (MAPK)/activator protein-1 (AP-1) and phosphorylation of NF-kb family protein (p50 and p52)-mediated pathways.^29–32^

### Characterization of CpG based DNA-MPs

**Fig. 2:**
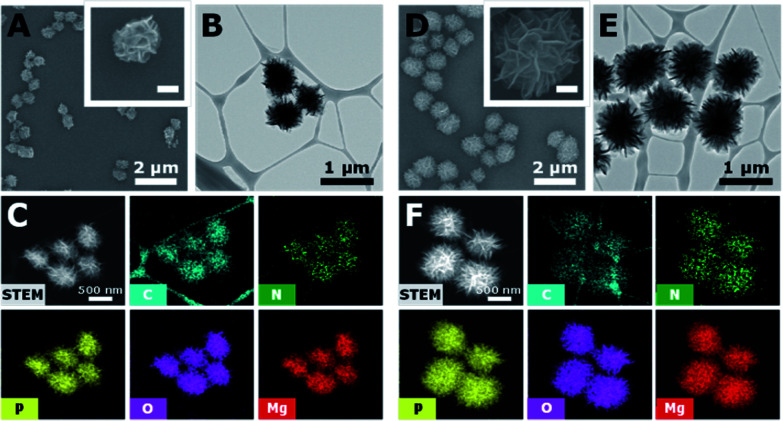
Characterization of the DNA-MPs. SEM images (A and D), TEM images (B and E), TEM based EDX-mapping images (C and F) of DNA-MP-0.5, (A–C) and DNA-MP-1.0, (D–F) indicating their spherical structures (inset scale bars in (A and D) 250 nm). TEM and TEM based EDX-mapping images show the compactly packed insides, morphologies and chemical compositions of the DNA-MPs.

To determine the size of the DNA-MPs favourable for macrophage uptake, we prepared DNA-MPs with different sizes by controlling the concentration of circular DNA (0.5 or 1 μM) and dNTP (0.8 or 2 mM). Interestingly, we could generate three different sizes of DNA-MPs with a diameter of 0.5, 0.8, and 1 μm by varying the concentration of circular DNA and dNTP (Fig. S1†). Among them, we chose DNA-MP-1.0 and DNA-MP-0.5 for testing their potential as efficient immune boosting adjuvants. The synthesis and morphology of the DNA-MPs were confirmed by SEM analysis. As shown in Fig. 2A and D, DNA-MP-0.5 and DNA-MP-1.0 were successfully fabricated at approximately 0.5 μm and 1.0 μm in average diameter, respectively. Previously, microsized DNA particles, *e.g.* hydrogels *via* a ligation method using x-shaped double stranded CpG strands, were developed with a broad range of particle sizes from 200 to 1000 nm.^33^ However, it should be noted that cRCA-based formulation of DNA-MPs resulted in greatly homogeneous microparticles with a narrow size distribution in this study. Furthermore, both of the DNA-MPs have spherical and densely compact structures composed of multiple sheets which could contribute to large surface areas for free access by enzymes. The overall morphologies were not changed when we synthesized DNA-MPs with a different template containing a GpC motif (Fig. S2†). In the TEM analysis, consistent results were observed as shown in the SEM results in terms of the size and the spherical structure of several sheets of the DNA-MPs. The TEM images also demonstrated that both of the DNA-MPs had densely packed insides (Fig. 2B and E). By TEM based energy-dispersive X-ray spectroscopy (EDX) mapping, we found that the DNA-MPs were composed of carbon (C), nitrogen (N), oxygen (O) and phosphorus (P) elements which are the construction components of DNA, indicating that the DNA-MPs were actually constructed with DNA (Fig. 2C and F). Taken together, these data indicate that a large amount of DNA could be compactly packaged into DNA-MPs using cRCA without any additional carrier.

### Release of CpGs from the DNA-MPs

**Fig. 3:**
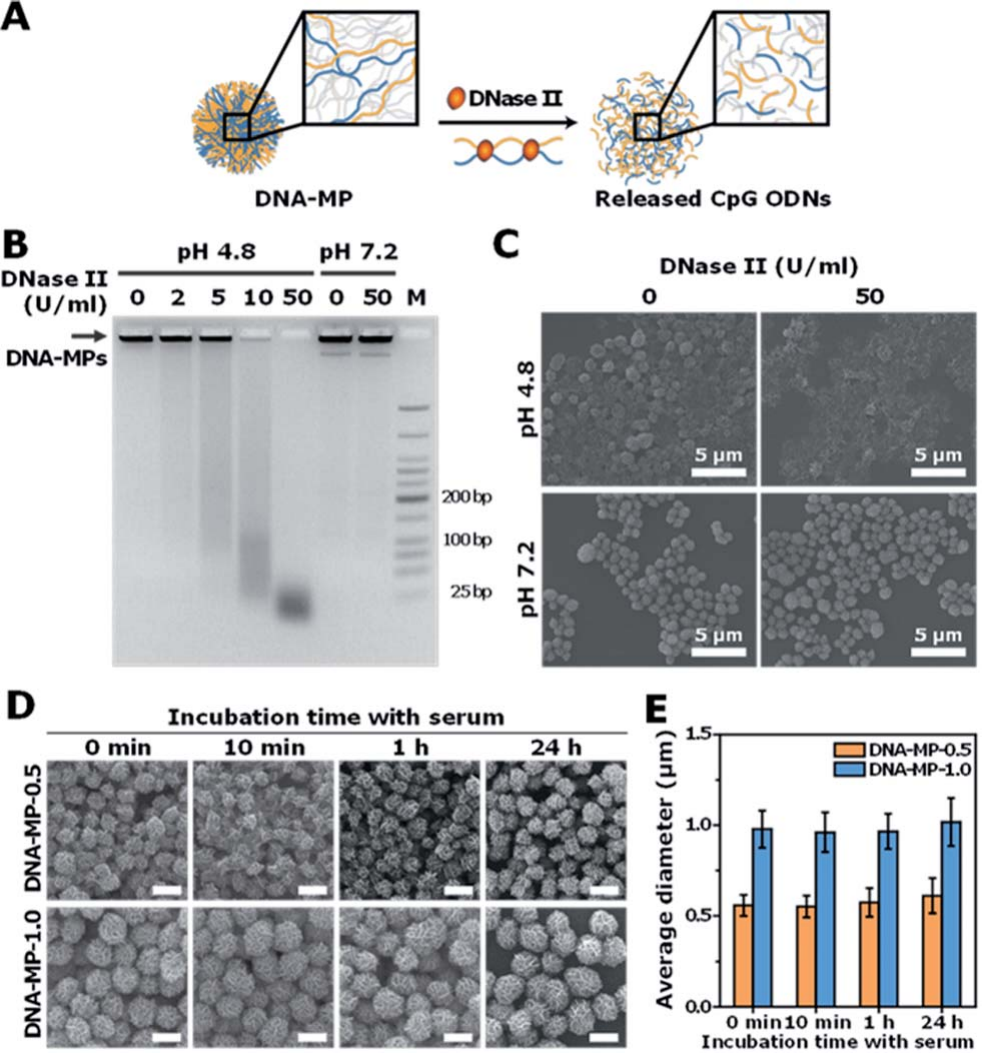
DNA-MPs releasing CpG ODNs mediated by DNase II and their stability under physiological conditions. (A) Schematic illustration of the release of CpG ODNs from the DNA-MPs mediated by DNase II. (B) Gel electrophoresis result of DNA-MP-1.0 cleaved by increasing the amount of DNase II at pH 4.8 and pH 7.2 for 4 h, respectively. The arrow indicates the DNA-MPs stuck in the loading well due to their large sizes. (C) SEM images of DNA-MP-1.0 (inset scale bars: 300 nm). (D) SEM images of the DNA-MPs treated with serum containing medium with respect to the incubation time (scale bars: 1 μm). (E) Average diameters of DNA-MP-0.5 (orange) and the DNA-MP-1.0 (blue) based on the analysis of SEM images using ImageJ software (*n* = 30).

To investigate the capability of the DNA-MPs as a CpG carrier, we further examined whether the DNA-MPs could release the CpG ODNs by DNase II treatment (Fig. 3A). Because DNase II is an abundant nuclease that exists in the phago-lysosomal compartment, it could mediate the release of CpG ODNs from the DNA-MPs by cleaving not single-stranded CpGs in the mismatched region, but double-stranded template DNA strands for successful interactions with TLR-9.^27,34^ For this reason, we incubated DNA-MP-1.0 with DNase II for 4 h at acidic pH (pH 4.8) and physiological pH (pH 7.2) to mimic the conditions inside of the phagolysosome and extracellular region/cytoplasm.^35,36^ After treatment of the DNA-MPs with several different concentrations of DNase II, the cleaved DNA-MPs were analyzed by gel electrophoresis. Fig. 3B shows that DNA-MP-1.0 was clearly cleaved into small pieces of DNA strands. As the amount of DNase II increased, the DNA-MPs were totally degraded into short strands of DNA at a high concentration of DNase II (50 U ml^−1^) at pH 4.8. At pH 7.2, on the other hand, DNA-MP-1.0 remained intact. The SEM image of DNA-MP-1.0 after treatment with DNase II reveals the disassembly of the overall construction of DNA-MP-1.0 at pH 4.8, indicating that acidic pH is the favorable condition for the release of CpG ODNs (Fig. 3C). It is noteworthy that CpG based DNA-MPs are resistant to DNase II-mediated degradation in physiological pH-like extracellular environments, while they are readily degraded at acidic pH, suggesting the possibility of release of CpG from the mismatched region of the particles only in the acidic phago-lysosomal compartment. Therefore, the accessibility of CpG ODNs with TLR-9 in the phago-lysosomal compartment, which is crucial for the following biological processing and immune stimulation, could be successfully achieved. To investigate the stability of the DNA-MPs under physiological conditions, we incubated the DNA-MPs with a 10% serum containing medium. As shown in Fig. 3D and E, the DNA-MPs maintained not only their constructions, but their sizes, until up to 24 h under serum conditions, indicating that both of the DNA-MPs might be resistant to degradation under physiological conditions. These results confirmed that the DNA-MPs could protect the CpG ODNs until they reach the phago-lysosomal compartment of the macrophages.

### Intracellular uptake of CpG based DNA-MPs

**Fig. 4:**
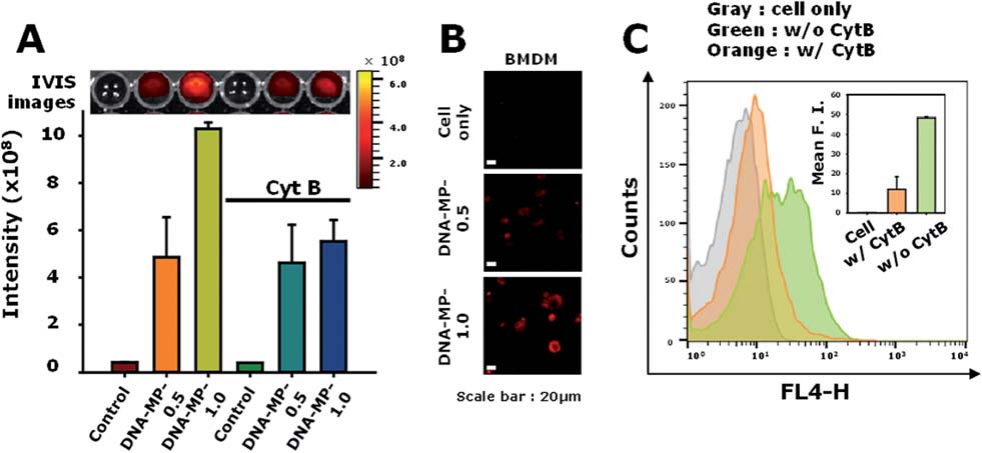
(A) Intracellular uptake of POPO3-labeled DNA-MP-0.5 and DNA-MP-1.0 to live RAW264.7 cells. (B) Confocal microscopy images of internalized POPO3-labeled DNA-MPs in the BMDMs. (C) FACS analysis of internalized Cy5-DNA-MP-1.0 in the absence and presence of cytochalasin B (cytB).

**Fig. 5:**
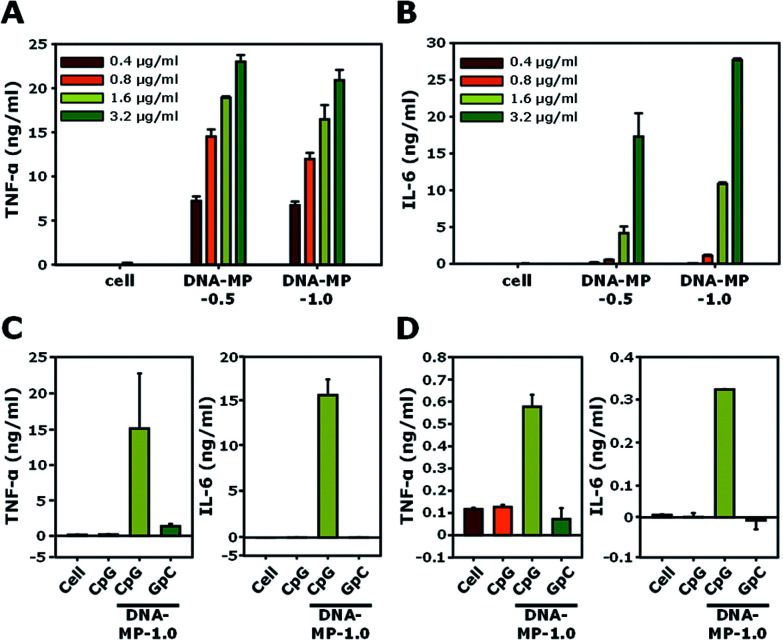
(A and B) The levels of released (A) TNF-α and (B) IL-6 in RAW264.7 cells treated with DNA-MP-0.5 and DNA-MP-1.0. (C) Released amounts of cytokines by the two types of DNA-MPs, CpG and GpC based microparticles in RAW264.7 cells. (D) Released amounts of TNF-α and IL-6 in the BMDMs.

Particle sizes are closely related to intracellular phagocytic uptake for APC-like macrophages and dendritic cells.^1,37^ In previous studies, hydrophobic polymeric particles with different shapes and sizes have been examined to determine optimal physicochemical properties for phagocytic activity.^37^ However, nucleic acid-based particles with hydrophilic and highly negatively charged characteristics have not yet been examined in terms of phagocytic activity for APCs in detail. To elucidate intracellular uptake efficiency regarding sizes of DNA particles, RAW264.7 cells were treated with DNA-MP-1.0 and DNA-MP-0.5. To monitor the DNA-MPs *in vitro*, the fluorescent dye POPO-3 was intercalated into the DNA-MPs before incubation with the cells. The extent of engulfed DNA-MPs was quantitatively analyzed by measuring fluorescence intensities within the cells (Fig. 4A). An approximately 2.5-fold higher fluorescence was observed in cells with DNA-MP-1.0, compared to those with DNA-MP-0.5. By treatment with actin polymerization inhibitor (cytochalasin B), the intracellular uptake by DNA-MP-1.0 was significantly decreased while DNA-MP-0.5 showed negligible change. To examine the intracellular uptake of the DNA-MPs for BMDMs, the BMDMs were quantitatively characterized by FACS analysis (Fig. S3†). More than 90% of the total cells were stained not with CD206 antibodies but with F4/80 antibodies. Confocal microscopy images in Fig. 4B show that much stronger red fluorescence signals were shown around the nuclei in cells with DNA-MP-1.0, compared to those with DNA-MP-0.5. The uptake of the DNA-MPs by the cells was also performed using Cy5 labeled DNA-MPs by preparation of DNA polymerization with Cy5-dCTP. As shown in Fig. S4,† the Cy5 intensity of Cy5-DNA-MP-1.0 was increased compared to that of non-labeled DNA-MP-1.0. Intracellular uptake and inhibition of intracellular uptake by cytochalasin B was also examined by FACS analysis after treatment with Cy5-DNA-MPs (Fig. 4C). The mean fluorescence intensities of the cells with PBS, DNA-MP-1.0, and DNA-MP-1.0 with cytochalasin B were 0.2 ± 0.1, 48.3 ± 0.4, and 12.0 ± 6.4, respectively. The mean fluorescence intensities of the DNA-MP-1.0-treated cells decreased down to 24.9% in the presence of cytochalasin B, as shown in Fig. 5C in the right panel. The Cy5-DNA-MPs allowed a much more significant suppression of particle uptake by cytochalasin B, compared to the fluorescence labeling of the DNA-MPs *via* physical intercalation. This result might be attributed to the nonspecific release of intercalated fluorescence dyes from the DNA-MPs during the experiments.

After internalization of the DNA-MPs *via* the phagocytic pathway, localization was examined by confocal microscopy (Fig. S5†). Early endosome antigen 1 (EEA1) is a well-known marker for the staining of early endosomes as well as early phagosomes.^38–40^ In this experiment, DNA-MP-1.0 was treated to cells and incubated with the cells for 8 h. In previous studies, microparticles with a size of 1–2 μm were seen to preferentially internalize into cells not by endocytosis, but by phagocytosis.^38,41^ Some DNA-MPs were co-localized with EEA1 probably due to localization in the early phagosomes. Others were shown by red-fluorescence in the cells, which might be due to their localization in late phagosomes (bottom panels of Fig. S5†).

### Cytokine induction by the DNA-MPs

The cell viabilities of DNA-MP-0.5 and DNA-MP-1.0 were examined using RAW 264.7 cells. In Fig. S6,† the DNA-MPs exhibit similar biocompatibility to that of free CpGs. Significant cytotoxicity was not observed up to a concentration of 20 μg ml^−1^. The biological effects of the DNA-MPs were examined by the level of two kinds of cytokines, TNF-α and IL-6, for RAW264.7 cells, as shown in Fig. 5A and B. After treatment of the two types of DNA-MPs at different concentrations (0.4, 0.8, 1.6, and 3.2 μg ml^−1^) to RAW264.7 cells for 8 h, the extent of the cytokines was quantitatively measured by ELISA. Upon increasing the amount of the DNA-MPs, the level of induced cytokines was elevated accordingly. A slightly higher amount of TNF-α was released from cells treated with DNA-MP-1.0, compared to those treated with DNA-MP-0.5. Fig. 5B shows a much higher induction of IL-6 in cells with DNA-MP-1.0 than those with DNA-MP-0.5. The levels of IL-6 in cells with DNA-MP-0.5 and DNA-MP-1.0 at a DNA concentration of 1.6 μg ml^−1^ were 4.1 ± 0.9 and 10.8 ± 0.2 ng ml^−1^, respectively. In previous studies, the released amount of IL-6 from cells treated with microsized CpG particles at a CpG concentration of 2.8 μg ml^−1^ (200 nM) was around 130 pg ml^−1^.^21,42^ Notably, CpG based DNA-MPs have a greater degree of biological activity in terms of IL-6 induction, which might be attributed to the mismatched structure of the CpG motif in the DNA-MPs and release of intact CpG sequences after DNase II cleavage in the phago-lysosomal region. Accordingly, DNA-MP-1.0, with its preferred biological activity, was utilized in the following experiment.

DNA-MP-1.0 showed a 2.6-fold and 1.6-fold higher induction of IL-6 than DNA-MP-0.5 at a DNA concentration of 1.6 and 3.2 μg ml^−1^, respectively. To investigate whether cytokine induction is in a sequence specific manner or not, CpG-based DNA-MP-1.0 and GpC-based DNA-MP-1.0 were treated to cells and cytokine induction was examined. As shown in Fig. 5C, DNA-MP-1.0 showed great induction of both TNF-α and IL-6, while no significant release of cytokines was observed in cells with GpC-based DNA-MPs. The levels of TNF-α inducted by CpG-based DNA-MP-1.0 were 83-fold and 11-fold higher than those of free CpG and GpC-based DNA-MP-1.0, respectively. The cytokine release experiment was also performed for primary BMDMs (Fig. 5D). The amounts of TNF-α and IL-6 inducted by CpG-based DNA-MP-1.0 were 570 ± 50 and 320 ± 0.5 pg ml^−1^, respectively. GpC-based DNA-MP-1.0 showed negligible effects on cytokine induction, similar to non-treated cells. Taken together, CpG-based DNA-MP-1.0 efficiently elicited cytokine release for RAW264.7 cells and BMDMs in a sequence specific manner. In previous studies, CpG delivery by carrier systems induced much lower amounts of IL-6 than TNF-α.^22,43^ The extent of IL-6 release by DNA-MP-0.5 was similar to that of previously reported 0.3 μm CpG based nanostructures, while DNA-MP-1.0 showed greatly enhanced IL-6 release compared to submicron sized 0.3 and 0.5 μm particles.^20^ Taken together, it should be also noted that CpG-based DNA-MP-1.0 exhibited particularly superior activity for IL-6 induction.

### Animal study

**Fig. 6:**
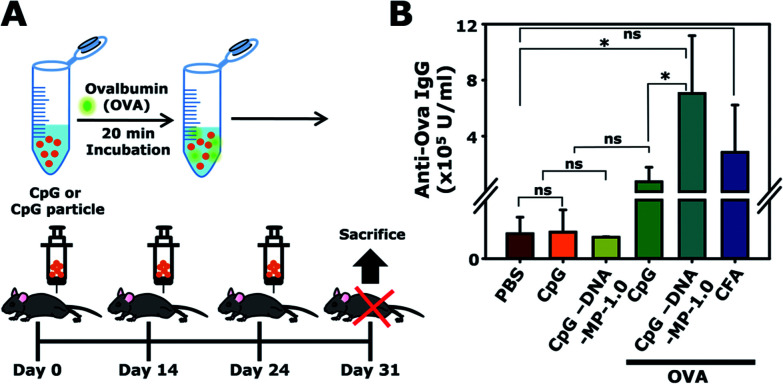
(A) Illustration of the immunization schedules for administration of DNA-MP-1.0 with OVA to C57BL/6 mice. (B) Quantification of the anti-OVA total IgG in mouse serum after intramuscular injection of different samples. *P < 0.05, ***P < 0.001; ns, not significant.

To examine whether DNA-MP-1.0 elicited a humoral cytokine induction and elevated target antibody production *in vivo*, CpG-based DNA-MP-1.0 was intramuscularly administered to mice three times, as shown in Fig. 6A. OVA was treated with CpG-based DNA-MP-1.0 as a model antigen. The amount of anti-OVA total IgG antibodies in serum was analyzed by ELISA (Fig. 6B). The levels of anti-OVA total IgG in samples of free CpG, DNA-MP-1.0, and CFA were 0.7 ± 1.0, 7.1 ± 4.1, and 2.8 ± 3.4 (× 10^5^ U ml^−1^), respectively. This result indicates that CpG-based DNA-MP-1.0 allowed greatly elevated antigen-specific antibody production *in vivo*, compared to free CpGs. Considering the superior serum stability of DNA-MP-1.0, it is conceivable that CpG-based DNA-MP-1.0 could be accumulated at injected sites in an intact form and engulfed by the APCs in muscle tissues. Notably, CpG-based DNA-MP-1.0 contains only DNA-based biomaterials without any additive materials, *e.g.* polymers and lipids, which might be good for strong immune stimulating activity without any toxicity. It is also worth noting that problems elicited by physicochemical stresses during encapsulation of functional CpG ODNs within carrier systems, *e.g.* degradation and physical aggregation by organic solvents, could be avoided. Thus, CpG-based DNA-MP-1.0 could contribute to effective immune stimulators for the delivery of therapeutic antigens. In addition, not only CpG ODNs, but also other functional nucleic acids, *e.g.* cDNA and mRNA, could be delivered by RCA-derived DNA-MP systems for antigen and immune stimulators for APCs.

## Conclusions

In this study, we developed elaborate adjuvant DNA-MPs by introducing a cRCA method for boosting immune stimulation without any additional carriers. Greatly homogeneous DNA-MP-1.0 provides a significantly improved level of uptake efficiency engulfed by the phagocytic pathway and ready release of free CpG ODNs, which provided sufficient cytokine secretion *in vitro* and antibody production in *in vivo* experiments. When compared to previous reports, this study not only presents a new direction for the release process of CpG ODNs from a mismatched structure in phagocytosis, but also provides a novel method for selective targeting macrophages by finely controlling the size of the particles. With this in mind, DNA-MP-1.0 could serve as a promising adjuvant for induction of humoral immune responses and the following production of selective antibodies without any noticeable toxicity *in vitro* and *in vivo*.

## Conflicts of interest

There are no conflicts to declare.

## Supplementary Material

RA-008-C7RA13293J-s001
