# Correction: CpG incorporated DNA microparticles for elevated immune stimulation for antigen presenting cells

**DOI:** 10.1039/c9ra90013f

**Published:** 2019-02-22

**Authors:** Heejung Jung, Dajeong Kim, Yoon Young Kang, Hyejin Kim, Jong Bum Lee, Hyejung Mok

**Affiliations:** Department of Bioscience and Biotechnology, Konkuk University 120 Neungdong-ro, Gwangjin-gu Seoul 05029 Republic of Korea hjmok@konkuk.ac.kr; Department of Chemical Engineering, University of Seoul 163 Seoulsiripdaero, Dongdaemun-gu Seoul 02504 Republic of Korea jblee@uos.ac.kr

## Abstract

Correction for ‘CpG incorporated DNA microparticles for elevated immune stimulation for antigen presenting cells’ by Heejung Jung *et al.*, *RSC Adv.*, 2018, **8**, 6608–6615.

In the published article there was an error in the primer DNA (22 nt) sequence in Table 1 on p. 6609. The correct sequence is shown in the table here below.

**Table 1:** Sequence information of naked CpG and linear DNAs for generating DNA-MPs. Naked CpG DNA, primers, linear DNAs for CpG, GpC, and their complementary strands. Blue: hybridization sites with primers; red: 20-base long CpG ODN; underlined red: CpG or GpC dinucleotide sites

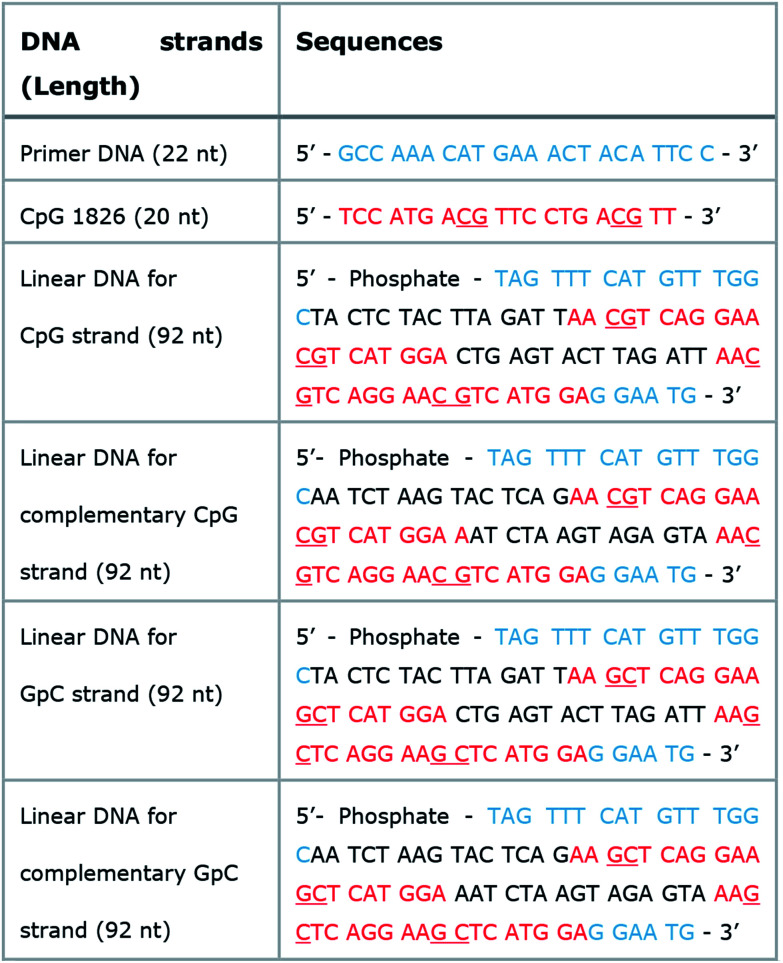

The Royal Society of Chemistry apologises for these errors and any consequent inconvenience to authors and readers.

## Supplementary Material

