# Supplementary material for: CpG incorporated DNA microparticles for elevated immune stimulation for antigen presenting cells
Source: RSC Adv. 2018 Feb 9;8(12):6608–15. doi: 10.1039/c7ra13293j (PMC9078369; doi:10.1039/c7ra13293j)
Supplement: RA-008-C7RA13293J-s001 [file RA-008-C7RA13293J-s001.pdf]

Electronic Supplementary Material (ESI) for RSC Advances.
This journal is © The Royal Society of Chemistry 2018

Supporting Information

CpG Incorporated DNA Microparticles for Elevated Immune Stimulation for Antigen Presenting Cells

Heejung Jung[1§], Dajeong Kim[2§], Yoon Young Kang[1], Hyejin Kim[2], Jong Bum Lee[2], and Hyejung Mok[1*]*

[1] Department of Bioscience and Biotechnology, Konkuk University, 120 Neungdong-ro, Gwangjin-gu, Seoul 05029, Republic of Korea

[2] Department of Chemical Engineering, University of Seoul, 163 Seoulsiripdaero, Dongdaemun-gu, Seoul 02504, Republic of Korea

§ The authors are equally contributed.

* Corresponding authors

tel: +82-2-450-0448, e-mail: hjmok@konkuk.ac.kr

tel: +82-2-450-0448, e-mail: jblee@uos.ac.kr

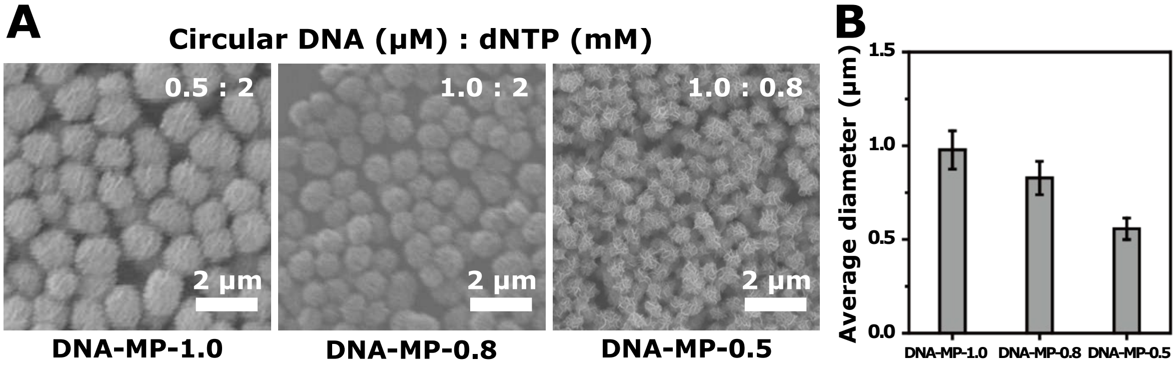

Fig. S1 A) SEM images and B) Average diameter of CpG-based DNA-MPs including DNA-MP-0.5, DNA-MP-0.8, and DNA-MP-1.0 prepared at different circular DNA: dNTP ratios.

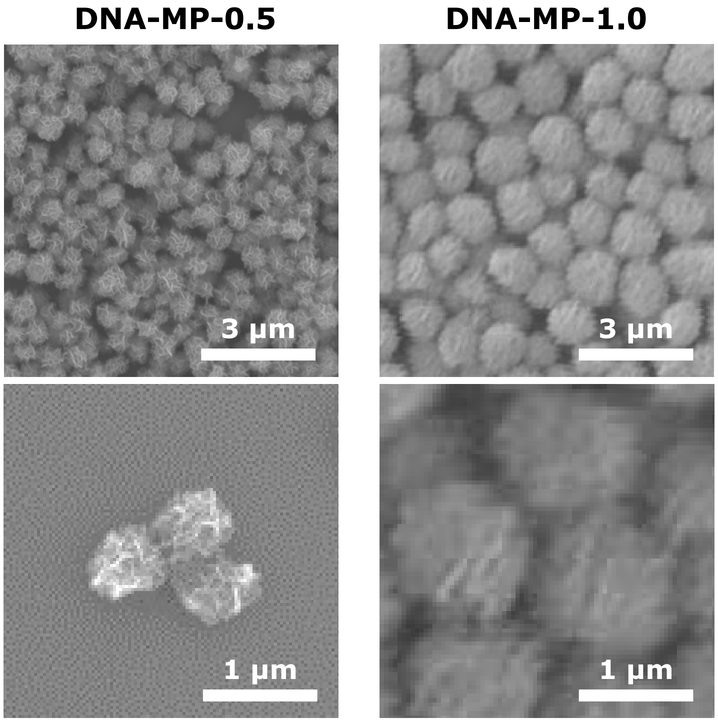

Fig. S2 SEM images of GpC-based DNA-MPs including DNA-MP-0.5 and DNA-MP-1.0.

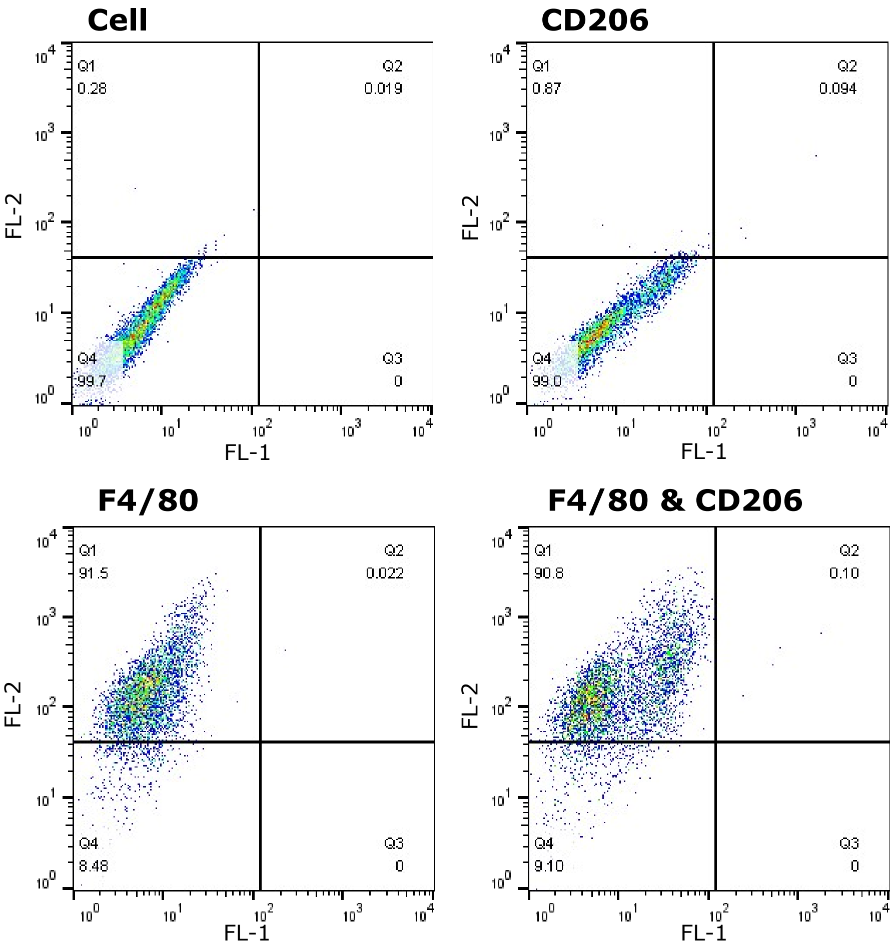

Fig. S3 FACS analysis of BMDMs after staining with PE-labeled F4/80 antibody and FITC-labeled CD-206 antibody.

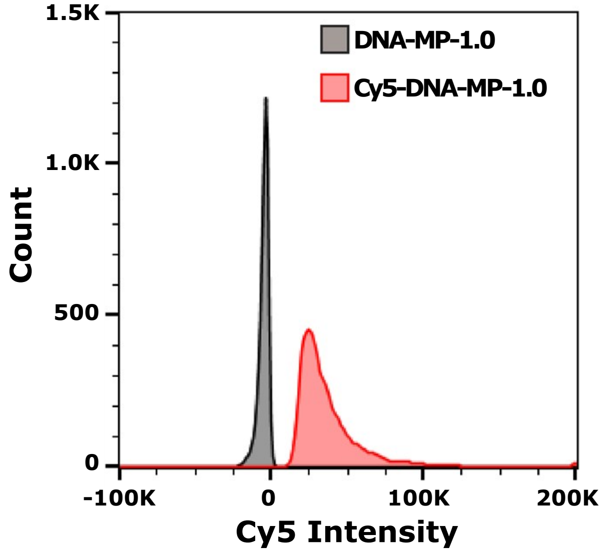

Fig. S4 Fluorescence intensities of Cy5 labeled DNA-MP-1.0, compared to non-labeled DNA-MP-1.0.

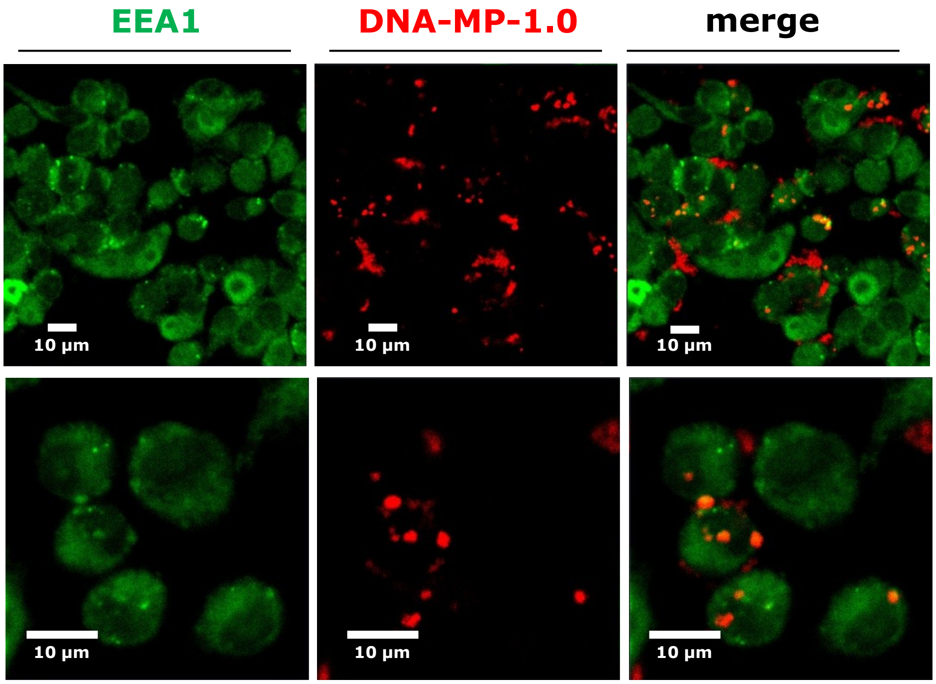

Fig. S5 Confocal microscopy images of cells treated with Cy5 labeled DNA-MP-1.0 after staining early endosome as well as early phagosome using FITC-labeled EEA1 antibodies.

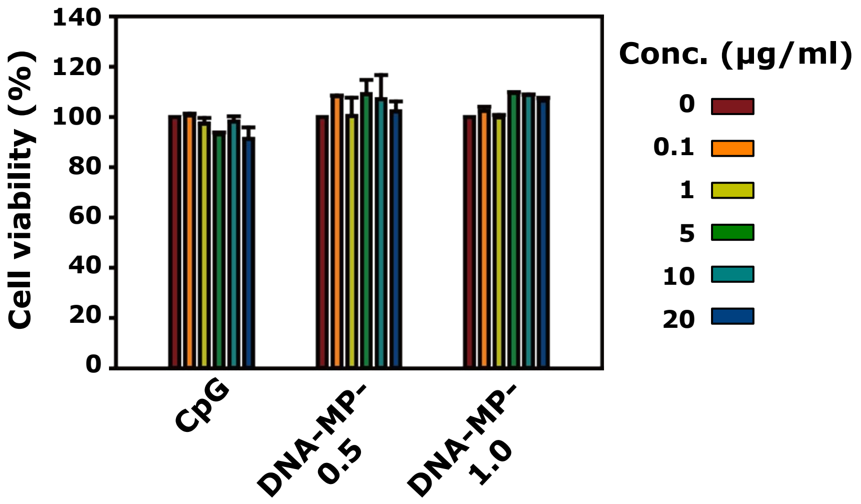

Fig. S6 Cell viability of DNA-MP-0.5 and DNA-MP-1.0 at different concentrations for RAW 264.7 cells.
